# Effects of Sex and 17 β-Estradiol on Cardiac Fibroblast Morphology and Signaling Activities In Vitro

**DOI:** 10.3390/cells10102564

**Published:** 2021-09-28

**Authors:** Kelsey Watts, William J. Richardson

**Affiliations:** Department of Bioengineering, Clemson University, Clemson, SC 29634, USA; kmwatts@clemson.edu

**Keywords:** cardiac fibroblasts, sex-specific, estrogen, fibrosis, heart failure

## Abstract

Several studies have demonstrated estrogen’s cardioprotective abilities in decreasing the fibrotic response of cardiac fibroblasts (CFs). However, the majority of these studies are not sex-specific, and those at the cellular level utilize tissue culture plastic, a substrate with a much higher stiffness than physiological conditions. Understanding the intrinsic differences between male and female CFs under more physiologically “healthy” conditions will help to elucidate the divergences in their complex signaling networks. We aimed to do this by conducting a sex-disaggregated analysis of changes in cellular morphology and relative levels of profibrotic signaling proteins in CFs cultured on 8 kPa stiffness plates with and without 17 β-estradiol (E2). Cyclic immunofluorescent analysis indicated that there was a negligible change in cellular morphology due to sex and E2 treatment and that the differences between male and female CFs occur at a biochemical rather than structural level. Several proteins corresponding to profibrotic activity had various sex-specific responses with and without E2 treatment. Single-cell correlation analysis exhibited varied protein–protein interaction across experimental conditions. These findings demonstrate the need for further research into the dimorphisms of male and female CFs to develop better tailored sex-informed prevention and treatment interventions of cardiac fibrosis.

## 1. Introduction

The prevalence of heart failure (HF) continues to rise, currently afflicting over 6.2 million Americans in roughly equal proportions among men and women [1,2]. What is not equal is the diagnosis, prognosis, treatment, and overall understanding of HF based on biological sex [2,3,4,5]. Female data are underrepresented in animal studies and clinical trials, so recommended treatment is not sex-specific, and adverse drug reactions occur at double the rate in women than in men [4]. Notably, premenopausal women have a relative protection against HF compared to age-matched men which subsides after menopause [2,6]. This phenomenon has been studied extensively and is largely thought to be because of the ovarian hormone estrogen [3,4,7]. Hormone replacement therapy (HRT) to maintain estrogen levels in postmenopausal women was even considered cardioprotective for several decades [8,9]. However, following randomized clinical studies, HRT was shown to have overall adverse trends, increasing the risk of stroke, breast cancer, and heart attack in postmenopausal women, and is not recommended for long-term use or as a preventive measure for cardiovascular diseases [6,8].

Although complete HRT is not a viable option to treat or prevent cardiac pathologies, 17 β-estradiol (E2) has exhibited promise in reducing cardiac fibrosis—an accumulation of collagens and other extracellular matrix components that reduces pump and electrical function [7,10,11,12]. After an initial myocardial infarction, a fibrotic response is necessary to maintain structural stability but can continue uncontrolled resulting in chronic HF [13]. There are currently no FDA-approved therapeutics to specifically target and control cardiac fibrosis [13]. In many in vitro studies, E2 treatment has been linked to a decreased fibrotic response of cardiac fibroblasts (CFs), indicating its potential as therapeutic [10,11,12,14,15,16]. It is important to note, many of these studies were carried out with neonatal rat CFs, pooling male and female cells together, so sex-specific effects of estrogen treatment were, for the most part, not investigated. Understanding how estrogen interacts with male and female cells at the molecular level is imperative to leverage estrogen’s therapeutic effects while minimizing potential adverse responses.

The few studies that do use sex-disaggregated analysis at the cellular level nearly all used tissue culture plastic (TCP) as the experimental platform. TCP has a stiffness that is magnitudes higher than physiologic conditions, even a fibrotic environment. CFs are extremely sensitive to their microenvironment and when cultured on stiff substrates many proteins become activated due to mechanotransduction pathways that can make cells profibrotic [17,18,19]. Additionally, a recent in vivo study by the Pinto group demonstrated the sex dimorphic response of the regulation of several genes within CFs due to angiotensin II stimulation [20]. Furthering our understanding of the intrinsic differences between male and female CFs under physiologically “healthy” conditions is a necessary first step to understanding the divergence of their intricate signaling pathways related to fibrosis. A substrate stiffness of 8 kPa was chosen for experiments because it is comparable to the stiffness of healthy myocardium and has been used to mimic a non-fibrotic environment in several other studies [21,22,23,24,25].

Expanding our knowledge of how estrogen interacts with both male and female CFs could aid in the discovery of novel treatment options for cardiac fibrosis that leverage estrogen’s cardioprotective properties while mitigating its harmful effects. In this study, we used cyclic immunofluorescence to investigate potential morphological changes, cellular localization, and activity levels of 12 proteins known to be heavily involved in estrogen and/or profibrotic signaling within CFs. This allowed for a sex-disaggregated analysis of not only each individual protein’s response to estrogen, but also single-cell cross-correlation analysis, which could uncover protein to protein crosstalk that could be potential sites to target for the regulation of cardiac fibrosis.

## 2. Materials and Methods

### 2.1. Cell Isolation and Culture

Adult Sprague Dawley rats (*n* = 8 male: 8 weeks, 265 g; and *n* = 8 female: 12 weeks, 255 g) were euthanized and hearts were removed and collected in Krebs-Henseleit buffer (Sigma, St. Louis, MO, USA). All procedures were performed with approval from Clemson University’s Institutional Animal Care and Use Committee (protocol AUP 2019-048). Ventricles were minced and digested to isolate CFs according to previously reported protocols [25,26]. Liberase TM (Roche, Indianapolis, IN, USA) was used in each of the six successive enzymatic digestions at 37 °C. Supernatants from each digestion were collected and centrifuged at 300× *g* and 4 °C and resuspended in Dulbecco’s Modified Eagle’s Medium (DMEM, Sigma) containing 10% fetal bovine serum (FBS, Atlanta Biologicals, Flowery Branch, GA, USA), 100 U/mL penicillin G, 100 µg/mL streptomycin, and 1 ng/mL amphotericin B (all Sigma). Following isolation, cells were plated in T-25 culture flasks and incubated at 37 °C and 5% CO_2_ for 4 h after which media was changed and, thereafter, was changed every 72 h until the serum starvation.

### 2.2. Collagen Coated Culture Plates

Prior to cell plating, 8 kPa 24-well CytoSoft^®^ plates (Advanced BioMatrix, San Diego, CA, USA) were coated with Telocol-3 bovine collagen (Advanced BioMatrix). Collagen solution was made at a 1:30 ratio of Telocol-3 in Phosphate Buffered Saline (PBS, Sigma). 1 mL of solution was pipetted into each of the 24 wells and allowed to polymerize at room temperature for 1 h. Excess solution was removed and the wells were washed with PBS twice.

### 2.3. Estrogen Treatment

Male and female CFs were passaged one time (P1) with 0.25% trypsin (Fisher, Hampton, NH., USA) at a 1:3 dilution before use in experiments. Once the CFs had reached ~75% confluence after the first passage, DMEM containing 10% FBS was removed, and flasks were washed with PBS. A 24 h serum starvation was started with phenol-free DMEM (Fisher) +2 mM l-glutamine (Fisher) and 2.5% charcoal-stripped FBS (GE Health, Chicago, IL, USA) incubated at 37 C and 5% CO_2_. After 24 h, CFs were passaged (P2) and plated onto the CytoSoft^®^ plates at 10,000 cells/well. CFs were divided into four experimental groups across two conditions: male vs. female and with or without 17 β-estradiol (E2, Sigma). The 24-well plates allowed for two biological replicates with three technical replicates (wells) per experimental condition. All wells were filled with 1 mL of phenol-free DMEM + 2 mM l-glutamine and 10% charcoal-stripped FBS. E2 was dissolved in ethanol at 10 mM, and 10 nM of E2 was added to wells designated for E2 treatment. An ethanol vehicle control of 10 nM was used as a control for all non-E2 treated wells. Plates were incubated at 37 °C and 5% CO_2_ for 24 h. Following incubation, all wells were fixed with 4% paraformaldehyde (PFA, Sigma) for 30 min and 99.9% methanol (Fisher) for 10 min. Immediately after fixation, plates were filled with PBS, wrapped in parafilm, and stored at 4 °C until use in cyclic immunofluorescence (CycIF).

### 2.4. Cyclic Immunofluorescence

Wells were washed with Odyssey blocking buffer (Fisher) for 1 h at room temperature on a rocker prior to antibody staining. Antibodies were purchased for the following proteins of interest: alpha-smooth muscle actin (α-SMA), filamentous actin (F-Actin), mothers against decapentaplegic homolog 3 (SMAD3), myocardin-related transcription factor (MRTF), nuclear factor of activated T Cells (NFAT), nuclear factor kappa-light-chain-enhancer of activated B Cells (NF-kB), phosphorylated extracellular signal-regulated kinase (p-ERK), phosphorylated focal adhesion kinase (p-FAK), phosphorylated jun n-terminal kinase (p-JNK), phosphorylated protein kinase B (p-Akt), phosphorylated p38 mitogen-activated protein kinase (p-p38), and rho-associated protein kinase (ROCK). Each antibody was individually optimized to determine unique staining dilutions and microscope gain, exposure, and light settings. Table S1 outlines where each antibody was purchased, Alexa Fluor conjugation, staining dilution, and microscope settings for each of the proteins of interest. The order of CycIF and protocol were determined according to published recommendations [27].

**Figure 1:**
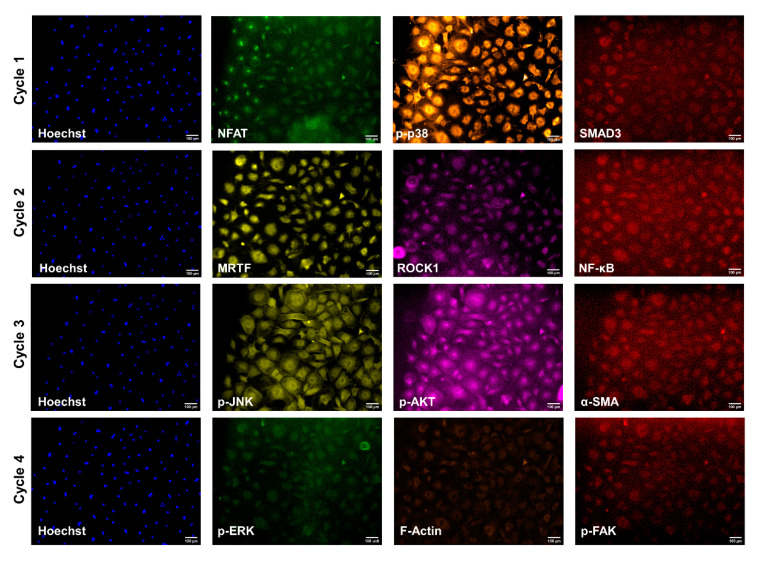
An example of a set of images for each of the proteins of interest for four rounds of CycIF. GFP, TxRed, and Cy7 (green, orange, and red) light cubes were used in rounds 1 and 4. RFP, Cy5, and Cy7 (yellow, pink, and red) light cubes were used in rounds 2 and 3. A Hoechst nuclear stain and DAPI light cube (blue) were used for all rounds.

Four consecutive rounds of CycIF were conducted with three proteins of interest in each round: (1) p-p38, NFAT, SMAD3, (2) MRTF, ROCK1, NF-kB, (3) p-JNK, p-Akt, α-SMA, and (4) F-Actin, p-ERK, p-FAK (Figure 1). Primary and Alexa Fluor conjugated antibodies were applied and rocked overnight at 4 °C. A secondary mouse-anti-rabbit IgG PE-Cy7 antibody for SMAD3, NF-kB, α-SMAD, and p-FAK was applied for 1 h at room temperature while rocking. A Hoechst nuclear stain was rocked for ten minutes at room temperature for each Cyc-IF round. All wells were washed four times with PBS between staining and imaging. Alexa Fluor light cubes GFP, TxRed, Cy7, and DAPI were used for rounds 1 and 4 of CycIF; RFP, Cy-5, Cy-7, and DAPI light cubes were used for rounds 2 and 3. The ThermoFisher Fluorescence Spectra Viewer was used to ensure minimal spectra overlap between channels [28]. An EVOS fluorescent microscope at 10× objective was used to take ten images per well, and beacons were saved to return to that position in consecutive CycIF rounds. Following each round of imaging, fluorophore inactivation was achieved by treating with 4.5% H_2_O_2_ (Fisher) in PBS plus 25 mM NaOH (Sigma) for 2 h under an LED light. Inactivation was confirmed visually with the EVOS before moving on to the next round of CycIF. Wells were washed with PBS four times after destaining and before the next round of CycIF. All images were saved as 8-bit TIFF files, which were imported into CellProfiler^TM^ for post-image processing [29].

### 2.5. Post-Image Processing

**Figure 2:**
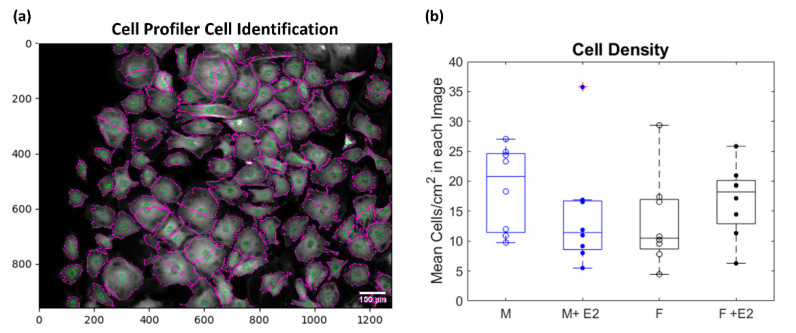
Sample CellProfiler^TM^ outlines of nuclei (green) and cells (purple) (**a**). A two-way ANOVA was used to determine if there was any significant interaction (α = 0.05) between sex (male = blue and female = black) and estrogen treatment (baseline = open dots to represent median of biological replicates and +E2 = closed dots) on cell viability. No significant difference existed within groups or interaction between groups for the mean cells/cm^2^ for each image (**b**).

In CellProfiler^TM^, the lower quartile intensity background was subtracted from each image. Images from consecutive rounds of CycIF were aligned with each other to account for small changes in the field of view that occurred over multiple rounds. The Hoechst nuclear stain images were used to identify Primary Objects (the nuclei) which were then used to identify Secondary Objects (cellular outlines) (Figure 2a). Morphological measurements of total cell area, nucleus area and location, and minor and major axis lengths were measured for each cell. To account for errors in automated cell identification, an upper bound of 10,000 µm^2^ and a lower bound of 1000 µm^2^ was set for acceptable cell areas. Integrated, mean, and median intensities were also recorded for each image channel (protein).

### 2.6. Statistical Analysis

Nearly 20,000 cells (~5000/experimental condition) were identified across the images taken from the eight male and female biological replicates and used to analyze morphological and protein-level data. Cell density was calculated per image across experimental conditions and there was no significant difference in cell viability with regards to sex or estrogen treatment (*p* > 0.05, Figure 2b). The median cell/nucleus area and elongation for each biological replicate were determined per experimental condition. To account for variability in fluorescent intensity among biological and technical replicates, normalization was conducted by dividing the channel (protein) intensity in each cell by the median of that channel intensity from all the cells on the entire plate (1 plate = 2 male and 2 female biological replicates). This allowed for comparison of relative protein levels across experimental conditions. Matlab’s anova2 function was used to run a two-way ANOVA to determine if a statistically significant (α = 0.05) difference existed between or within groups of sex (male vs. female) and estrogen treatment (baseline and +E2). Box and whisker plots which show the median, 25th, and 75th percentiles of the eight biological replicates per experimental condition were generated; if an outlier was determined to be present it is denoted by a red cross over the biological replicate. When there were instances of statistical significance it is denoted on the graph and all *p*-values are reported in Table S2. Single-cell correlation coefficients for each protein–protein and protein–morphology interaction were also calculated using MATLAB’s built-in corrcoef function.

## 3. Results

### 3.1. Sex-Dissagregated Analysis of CF Morphology

**Figure 3:**
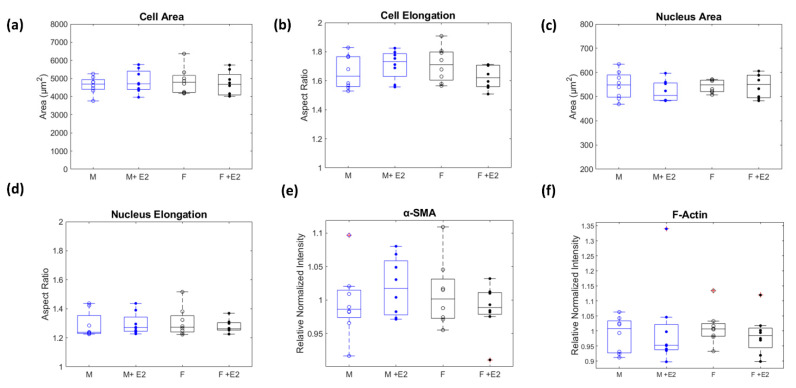
A two-way ANOVA was used to determine if there was any significant interaction (α = 0.05) between sex (male = blue and female = black) and estrogen treatment (baseline = open dots to represent median of biological replicates and +E2 = closed dots) on morphological factors. No significant difference existed within groups or interaction between groups for total cell area and elongation (**a**,**b**), nor nucleus area and elongation (**c**,**d**). F-Actin and α-SMA relative protein levels did not significantly change between experimental conditions (**e**,**f**).

Microscopic image analysis demonstrated no change in cell area across experimental conditions (*p* > 0.05, Figure 3a). Likewise, cell elongation (*p* > 0.05, Figure 3b), which was calculated by determining each cell’s aspect ratio (major/minor axis), was also not affected by sex or estrogen treatment. Nuclear area and aspect ratio were observed and determined not to be dependent on sex or estrogen treatment (*p* > 0.05, Figure 3c,d). F-Actin and α-SMA’s relative protein concentrations (*p* > 0.05, Figure 3e,f) did not vary among experimental conditions, indicating that under physiological like conditions, the structure and morphological presentation of male and female CFs do not vary significantly.

### 3.2. Relative Levels of Fibrotic Related Signaling Proteins

**Figure 4:**
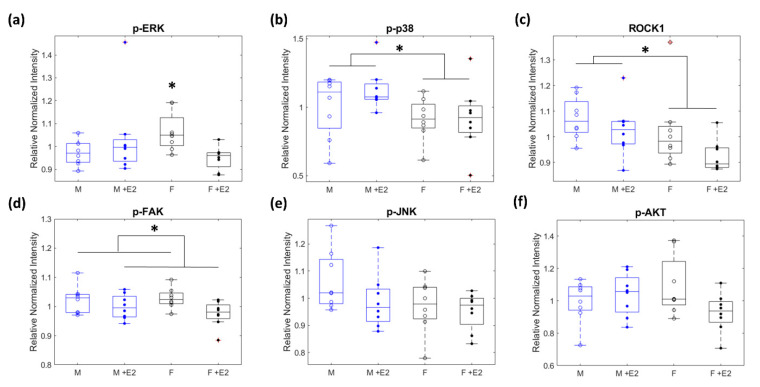
A two-way ANOVA was used to determine if there was any significant interaction (α = 0.05) between sex (male = blue and female = black) and estrogen treatment (baseline = open dots to represent median of biological replicates and +E2 = closed dots) on the normalized median intensity of profibrotic proteins. p-ERK showed a significant interaction between sex and E2 treatment (* *p* < 0.05, (**a**)). The median intensity of ROCK1 and p-p38 was significantly different due to biological sex (* *p* < 0.05, (**b**,**c**)). E2 treatment caused a significant difference in median intensity for p-FAK (* *p* < 0.05, (**d**)). No significant interactions between or within groups existed for p-JNK or p-AKT (**e**,**f**).

Relative protein levels were determined by comparing normalized median cell intensities for each protein of interest. p-ERK had a statistically significant interaction between sex and E2 treatment with the female baseline being higher than all other experimental conditions (*p* < 0.05, Figure 4a). p-p38 and ROCK1 were found to be statistically different due to sex, with male cells having higher levels of both p-p38 and ROCK1 in the baseline and E2 treated cells compared to female cells with or without E2 (*p* < 0.05, Figure 4b,c). p-FAK showed a statistically significant downregulation of the relative levels of p-FAK in both male and female cells when E2 was present (*p* < 0.05, Figure 4d). No statistically significant change existed across experimental conditions for the relative protein levels of p-JNK and p-Akt (*p* > 0.05, Figure 4e,f).

### 3.3. Nuclear Localization of Mechanosensitive Proteins

**Figure 5:**
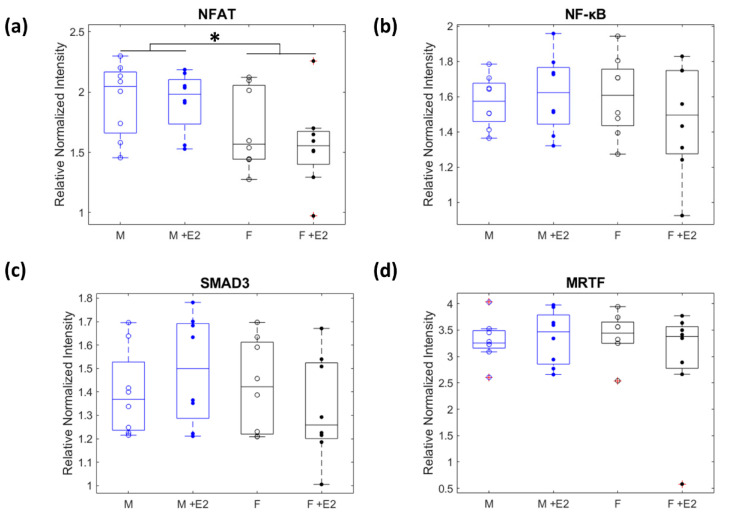
A two-way ANOVA was used to determine if there was any significant interaction (α = 0.05) between sex (male = blue and female = black) and estrogen treatment (baseline = open dots to represent median of biological replicates and +E2 = closed dots) of translocation of profibrotic proteins to the nucleus. The median nucleus: cytoplasm ratio of NFAT was significantly different due to biological sex (* *p* < 0.05, (**a**)). There were no significant interactions between or within groups for NF-κB, SMAD3, or MRTF (**b**–**d**).

Many profibrotic proteins in CFs are in their most activated form when they have translocated to the nucleus, which allows them to act as transcription factors to influence gene regulation. In our study, MRTF, NFAT, NF-κB, and SMAD3 are most activated in the nucleus. Therefore, instead of measuring their total cell intensity, we calculated the ratio of the intensity within the nucleus vs. the cytoplasm (normalized mean nuclear intensity/normalized mean cytoplasm intensity). While MRTF, NFAT, NF-κB, and SMAD3 all had ratios greater than 1 for each experimental condition indicating that more was present in the nucleus than the cytoplasm, only the levels of NFAT were different across experimental groups (*p* > 0.05, Figure 5a–c). Male cells had NFAT levels in the nucleus that were higher than both the baseline and E2 treated female cells (*p* < 0.05, Figure 5d).

### 3.4. Correlation Analysis of Protein-Protein Interactions

**Figure 6:**
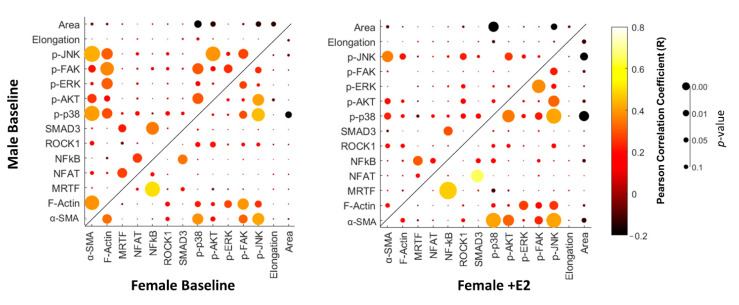
Dot plots of correlation coefficients and their corresponding *p*-values for analysis of protein–protein and protein–morphology interactions.

An advantage of cyclic-IF analysis for protein quantification is that it enables single-cell measurements, which can be tested for protein–protein and protein–morphology relationships. The Pearson’s correlation coefficients of the normalized relative protein–protein levels and protein–morphology interactions were calculated along with their corresponding *p*-values. These data were used to create dot plots (Figure 6), which allow for comparison of changes in protein–protein/protein–morphology interactions between experimental conditions. The most striking difference is that a much stronger correlation of protein–protein interactions for female CFs treated with estrogen (indicated by large orange and yellow dots) existed than for male CFs treated with estrogen. Similarly, male CFs without E2 demonstrated a number of strong and significant correlations, which were dampened in the presence of E2. Female CFs experienced similarly correlated relationships with and without E2 treatment.

## 4. Discussion

Although many studies note the phenotypic differences between male and female cardiac fibroblasts, very few have investigated if these phenotypic changes result in observable morphological differences in cell size and elongation. At a macro level, male and female morphology are dimorphic, with male hearts and their components, including the left ventricle, often being larger than female hearts from the same species [30,31]. As fibrosis progresses, CFs undergo morphological changes, elongating and covering a larger area due to interactions with their changing microenvironment [24]. This can also cause nuclear morphologic changes mediated by LINC [19]. To fully understand the differences in how male and female CFs interact with and respond to changes in their mechanochemical environment during fibrosis progression, it is necessary to know if any morphological differences are present in physiologically “healthy” environments. Our results indicate that on a stiffness that mimics physiological conditions, there are no changes in cell and nuclear morphology due to sex and estrogen treatment. This indicates that while male and female cells may be phenotypically different at an intracellular level, these changes are more likely to present biochemically rather than structurally. Our finding of no significant difference in α-SMA and F-Actin relative protein levels due to sex and estrogen treatment also supports this theory, as elevated α-SMA and F-Actin levels are both indicative of increased cell contractility, which can cause changes in cell morphology [32].

Of the 12 proteins investigated, NFAT, p-p38, and ROCK1 were found to be more elevated in male cells than female cells regardless of E2 treatment. Each of these proteins is typically more elevated in a profibrotic environment [19]. This indicates that even in a physiologically “healthy” environment, male CFs may be more sensitive to chemical changes and prone to fibrotic behavior than female CFs. The sex disaggregated literature of the behavior of these proteins in relation to fibrosis in CFs is extremely sparse. One in vivo mouse study found that female mice underwent p-38-induced ventricular hypertrophy and mortality at a slower rate than male mice [33]. Future research should investigate potential intrinsic differences of NFAT, p-p38, and ROCK1 and other downstream proteins in male and female CFs to clarify the divergence of male and female signaling pathways. This could support the development of sex-specific prevention and treatment methods for cardiac fibrosis.

CFs are very susceptible to changes in their microenvironment. A major way they sense and translate these signals within the cell is through integrins and adhesion receptors on the cell membrane. One highly studied CF adhesion receptor is focal adhesion kinase (FAK), which can be activated (p-FAK) by interactions with the extracellular matrix [24]. In numerous studies, FAK inhibition has been shown to stop adverse cardiac remodeling [34,35]. Our results showed that upon treatment with E2, both male and female CFs had reduced expression of p-FAK, indicating its promise as a potential regulation pathway that mimics estrogen’s cardioprotective effect. To our knowledge, no other studies investigate the effect of estrogen on FAK in cardiac fibroblasts. However, there are a few studies that demonstrate how E2 treatment can activate FAK in breast cancer cells [36,37]. The microenvironment of a breast cancer tumor is likely much stiffer than the 8 kPa physiological-like stiffness used in our study, so it is possible that there is a complex interaction of mechanical cues and hormone signaling which affect FAK activation. FAK has many proteins downstream of it which are also considered profibrotic factors, so FAK’s pathways are a promising source of potential regulation if more research is conducted to understand its response to combined estrogen treatment and mechanical stimulus.

Not all of our proteins of interest had statistically significant differences between experimental conditions (SMAD3, NF-κB, p-JNK, and p-Akt). This finding was slightly surprising with regards to SMAD3 and p-JNK, because of the past literature that cites the ability of estrogen to downregulate SMAD3 and p-JNK activity in CFs [7]. These contradictory findings are not unusual—a recent review of the limited research of sex differences and estrogen signaling in CFs notes additional discrepancies among various other peer-reviewed studies [7]. There are many differences in experimental set up including in vivo vs. in vitro design, pooled male and female cells vs. sex disaggregated analysis, and neonatal vs. adult cells. Our study adds an additional variable, substrate stiffness. Nearly all previous in vitro studies of sex or estrogen signaling in CFs were carried out on TCP which has an unrealistically high stiffness (>1000 fold stiffer than myocardium). It is imperative to conduct further sex/E2 focused studies within CFs controlling for individual variables before it is possible to synthesize the results from multiple studies into a broader understanding of sex-specific and estrogen-induced signaling in CFs.

In our study, the only protein of interest that had a statistically significant interaction between sex and E2 treatment was p-ERK. Baseline levels of p-ERK in female CFs were higher than in any other experimental condition; however, upon E2 treatment, female CFs had levels similar to male CFs. There was a negligible difference between the male baseline and male +E2 relative protein levels of p-ERK. We hypothesize that this difference among experimental conditions may related be to β-Adrenergic receptors (β-ARs), which are believed to increase fibrotic activity through ERK(1/2) related pathways [38]. Many studies have observed crosstalk in β-ARs and estrogen signaling [39]. Additionally, a recent study outlined the sex dimorphic response in CFs due to β-AR stimulation [40]. As β-blockers are already an FDA-approved treatment for many cardiovascular pathologies, including high blood pressure and heart failure, this connection offers a promising avenue of potential regulation of uncontrolled fibrosis that warrants further investigation.

Limitations of our study include that it was simply an in vitro monolayer culture analysis with a serum starvation used to induce the baseline lack of estrogen condition. In the future, enhanced in vitro platforms that utilize (1) a coculture of the multiple cell types present in the myocardium, (2) a 3D cell culture platform such as hydrogels, and (3) applying cyclic stretch, could all be used to better mimic a healthy cardiac environment [25,41,42,43,44,45,46]. We also want to acknowledge that α-SMA expression remained elevated in our cells during the short experimental period. This is likely due to mechanical memory wherein cells become activated during initial plating on TCP then maintain some of that activity even after reseeding onto softer substrates [47]. Future studies will either be conducted for a longer time course or cultured on softer substrates immediately after cell isolation. Additionally, an in vivo study with OVX mice could be used to truly mimic the changes in estrogen levels due to menopause and other differences that are difficult to capture with an in vitro platform. We also chose to use immunofluorescence to capture any potential morphological and nuclear translocation of profibrotic factors intrinsic to male and female CFs with and without estrogen treatment. Our analysis indicated that no significant structural differences existed between male and female CFs on a physiologically similar stiffness of 8 kPa, and only NFAT expressed different levels of translocation to the nucleus among experimental conditions. In future research, we would recommend that analysis could be carried out with methods that could allow for a more robust signaling analysis such as flow cytometry, Western blotting, or RNA-seq.

A more robust data set would provide the opportunity to conduct in silico experiments which could further elucidate our understanding of the complex signaling networks of CFs. Our study was primarily focused on how mechanically activated signaling pathways in CFs are impacted by estrogen and biological sex. There are other profibrotic and proinflammatory pathways (i.e., DAMPs) in CFs independent of mechanical stimulation that may be affected by biological sex and/or estrogen which warrant study [48]. In addition, the downstream response to estrogen stimulation can be affected by the presence of estrogen receptors (ER-α, ER-β, and GPR30) [49,50]. Future studies should utilize a sex-disaggregated analysis to uncover possible differences in estrogen receptor expression and regulation under physiologically “healthy” conditions. A computational approach will facilitate the synthesis of findings from many independent experiments into a network of the complex interactions of cardiac fibroblast signaling.

## 5. Conclusions

Our results support the existing literature that cites male and female CFs are sexually dimorphic, even under physiologically “healthy” conditions, and should be treated as such when designing experiments to allow for sex-disaggregated analysis to determine how biological sex may affect the response to treatment interventions. Future research could be directed toward uncovering the complex signaling interactions related to biological sex, E2, and profibrotic signaling pathways. One way to hasten this investigation could be through the use of sex-specific computational disease models. Existing disease models such as the signaling network model of cardiac fibroblasts’ response to mechano-chemo signaling could be improved through the incorporation of biological sex and hormone pathways [51,52]. Large-scale sex-specific network modeling could greatly accelerate the pace and reduce the costs of identifying important interactions involved in the regulation of fibrosis rather than trial and error experiments alone.

## Data Availability

All data produced by this study will be available through the Richardson lab figshare account: https://figshare.com/account/home#/projects/121860.

## Supplementary Materials

The following are available online at https://www.mdpi.com/article/10.3390/cells10102564/s1, Table S1: Outline of four rounds of CycIF including antibodies used, Alexa Flour conjugation, dilution ratio, and microscope settings (Light, Exposure, and Gain), Table S2: All p-values from two-way ANOVA analysis. Significance at α = 0.05 are bolded and denoted with an *.
