# Supplementary material for: Effects of Sex and 17 β-Estradiol on Cardiac Fibroblast Morphology and Signaling Activities In Vitro
Source: Cells. 2021 Sep 28;10(10):2564. doi: 10.3390/cells10102564 (PMC8533711; doi:10.3390/cells10102564)
Supplement: Supplementary file 1 [file cells-10-02564-s001.zip › cells-1383171-supplementary.pdf]

Table S1. Outline of four rounds of CycIF including antibodies, used, Alexa Flour conjugation, dilution ratio, and micro-scope settings (Light, Exposure, and Gain).

CycIF Round	Antibody	Manufacture	Alexa Flour	Dilution	Light	Exposure	Gain
1	Anti-p-p38 (E-1)	Santa Cruz (sc-166182 AF594)	594 (Tx-Red)	1:25	65	703	13.3
	Anti-NFATc4 (B-2)	Santa Cruz (sc-271597 AF488)	488 (GFP)	1:30	61	462	12.2
	Anti-Smad3 (38-Q)	Santa Cruz (sc-101154)	*	1:25	52	755	15.2
2	Anti-MRTF-A (G-8)	Santa Cruz (sc-390324 AF546)	546 (RFP)	1:25	52	582	6.6
	Anti- Rock-1 (G-6)	Santa Cruz (sc-17794 AF647)	674 (Cy5)	1:30	61	586	14.3
	Anti-NFκB p50 (E-10)	Santa Cruz (sc-8414)	*	1:25	58	908	15.4
3	Anti-p-JNK (G-7)	Santa Cruz (sc-6254 AF546)	546 (RFP)	1:50	42	151	7.4
	Anti-p-Akt1/2/3 (C-11)	Santa Cruz (sc-514032 AF647)	647 (Cy5)	1:30	61	586	14.3
	Anti-ASMA	abcam (ab5694)	*	1:250	61	911	14.4
4	Anti-p-ERK (E-4)	Santa Cruz (sc-7383 AF488)	488 (GFP)	1:30	58	391	14
	Texas Red®-X phal-loidin	Fisher (T7471)	594 (Tx-Red)	1:40	65	676	17.1
	Anti-p-FAK (Tyr397)	invitrogen (700255)	*	1:250	58	1140	15.4
1 - 4	*mouse-anti-rabbit IgG PE-Cy7	Santa Cruz (sc-516721)	750 (Cy7)	1:100	-	-	-
	Hoechst 33342	Fisher (H3570)	DAPI	1:1000	35	51	9

Table S2. All p-values from two-way ANOVA analysis. Significance at α=0.05 are bolded and denoted with an *. .

	p_{E2}	p_{sex}	p_{sex*E2}
**Cell Density**	0.87	0.58	0.15
**Cell Area**	0.89	0.81	0.43
**Cell Elongation**	0.62	0.67	0.07
**Nucleus Area**	0.45	0.53	0.43
**Nucleus Elongation**	0.72	0.97	0.49
**α-SMA**	0.94	0.57	0.16
**F-Actin**	0.92	0.97	0.49
**P-ERK**	0.49	0.94	**0.02***
**P-p38**	0.37	**0.04***	0.43
**ROCK1**	0.06	**0.048***	0.47
**P-FAK**	**0.01***	0.53	0.34
**p-JNK**	0.17	0.07	0.394
**p-AKT**	0.24	0.86	0.06
**NFAT**	0.52	**0.01***	0.62
**NF-κB**	0.63	0.50	0.30
**SMAD3**	0.99	0.38	0.20
**MRTF**	0.43	0.53	0.31
